# Fluorine-18 ImmunoPET Imaging of Antibody Brain Kinetics and Amyloid-Beta Pathology

**DOI:** 10.1021/acsptsci.5c00359

**Published:** 2025-07-11

**Authors:** Eva Schlein, Sara Lopes van den Broek, Tiffany Dallas, Ken G. Andersson, Stina Syvänen, Jonas Eriksson, Dag Sehlin

**Affiliations:** † Department of Public Health and Caring Sciences, 8097 Uppsala University, 751 85 Uppsala, Sweden; ‡ 690629 BioArctic AB, 112 51 Stockholm, Sweden; § Department of Medicinal Chemistry, Uppsala University, 751 23 Uppsala, Sweden; ∥ PET Centre, Uppsala University Hospital, 751 85 Uppsala, Sweden

**Keywords:** positron emission tomography (PET), bispecific antibody, receptor-mediated transcytosis (RMT), blood–brain barrier (BBB), Alzheimer’s disease (AD), amyloid-β (Aβ)

## Abstract

Bispecific antibodies utilizing the transferrin receptor (TfR) for transport into the brain are being developed for both therapeutic and diagnostic targeting of the amyloid-β (Aβ) protein that deposits in the Alzheimer’s disease (AD) brain. In contrast to traditional antibodies, TfR-binding bispecific antibodies display rapid and efficient brain uptake. However, due to differences in pharmacokinetic properties, it has been challenging to directly compare mono- and bispecific antibody brain uptake in vivo. Here, we have studied the Aβ antibody Bapineuzumab (Bapi) and its bispecific variant Bapi-Fab8D3, which contains a fragment of the TfR-binding antibody 8D3, enabling receptor-mediated transcytosis into the brain. Both antibodies were engineered to reduce binding to the neonatal Fc receptor (FcRn), thereby increasing their clearance from the blood. The antibodies were radiolabeled with fluorine-18 (^18^F) and administered to wildtype (WT) mice, which were PET scanned in an alternating manner to visualize antibody brain pharmacokinetics over a period of 9 h, followed by ex vivo analyses. Next, the bispecific antibody [^18^F]­F-Bapi-Fab8D3^FcRn–^ was used for PET imaging to quantify Aβ pathology in AD mouse model *App* ^*NL‑G‑F*^ mice at 12 h after antibody administration. [^18^F]­F-Bapi ^FcRn–^ and [^18^F]­F-Bapi-Fab8D3^FcRn–^ had identical blood concentration curves in the WT mice. PET data quantification demonstrated that while the brain concentration of [^18^F]­F-Bapi^FcRn–^ declined, that of [^18^F]­F-Bapi-Fab8D3^FcRn–^ increased throughout the 9 h time period, indicative of its active transport into the brain. PET imaging discriminated *App* ^*NL‑G‑F*^ from WT mice at 12 h after [^18^F]­F-Bapi-Fab8D3^FcRn–^ administration, suggesting that this novel antibody-based tracer may be used for the same-day PET imaging of Aβ.

## Graphical abstract

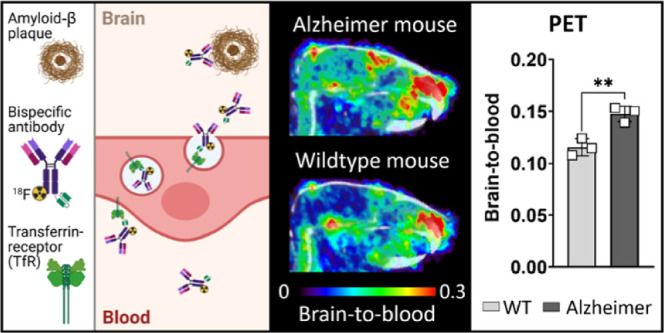

Protein drugs make up a growing class of therapeutics being introduced to the market. The majority of protein drugs are antibodies directed toward various targets related to cancer or autoimmune disease. Historically, it has been difficult to develop antibody therapeutics for brain diseases, primarily due to the blood–brain barrier (BBB), which prevents macromolecules such as antibodies from entering the brain. However, recent progress in Alzheimer’s disease (AD) drug development has resulted in two antibodies being approved by the US Food and Drug Administration (FDA), *lecanemab* and *donanemab*, both targeting pathological amyloid-β (Aβ) aggregates.^1–4^ Along with the development of drugs targeting Aβ, there has been an increased use of diagnostic methods to detect and quantify Aβ, both for inclusion of patients in clinical trials and to evaluate the effects of treatment. Positron emission tomography (PET) imaging with amyloid ligands such as [^11^C]­PiB detects Aβ plaques in the brain of AD patients and is a widely used method in clinical trials. However, current amyloid ligands detect only the dense amyloid core of Aβ plaques, while therapeutic Aβ antibodies primarily bind to diffuse Aβ aggregates in the outer rim of the Aβ plaques. This discrepancy increases the risk of misdiagnosis and potentially flawed interpretation of drug effects in clinical trials. A better strategy could be to directly use radiolabeled antibodies as ligands for PET imaging and detection of Aβ deposits in the AD brain.^5^

Antibody-based PET imaging (immunoPET) is under development for various types of tumors. However, the BBB poses a challenge in using antibody ligands for brain immunoPET. A potential solution is to employ the “molecular Trojan horse” strategy, in which a targeting antibody is fused to a molecule that binds to an endogenous receptor capable of transporting the antibody across the BBB through receptor-mediated transcytosis (RMT).^6,7^ We have previously developed a number of bispecific antibody formats targeting Aβ in combination with the transferrin receptor (TfR), which is the most commonly used receptor for RMT. When radiolabeled, these bispecific antibody ligands can be used for immunoPET imaging of brain Aβ in mice that develop Aβ pathology. We have thus shown that, compared with the traditional amyloid ligand [^11^C]­PiB, immunoPET can detect Aβ at an earlier stage and with higher accuracy detect the therapeutic effects of Aβ lowering treatment.^8–13^ However, because antibody clearance from both blood and brain is a slow biological process, these ligands often require a prolonged interval between injection and PET imaging to achieve sufficient contrast. This necessitates the use of relatively long-lived radioisotopes and higher dosing. Antibodies are kept in circulation through an interaction with the neonatal Fc receptor (FcRn), which prevents the clearance of antibodies from the blood. By introducing mutations to the Fc domain of an antibody, its biological half-life can be reduced,^14^ which could be beneficial in the context of immunoPET imaging.

Here, we have evaluated an antibody pair based on the humanized Aβ antibody Bapineuzumab (Bapi): one of them is a standard monospecific IgG (Bapi) and the other a bispecific, brain-penetrating antibody (Bapi-Fab8D3), both engineered with a mutation to reduce binding to the FcRn. In a previous study, we demonstrated that both antibodies exhibited a drastically reduced half-life in the blood. Moreover, while Bapi^FcRn–^ showed minimal brain uptake and retention in Aβ expressing mice over time, Bapi-Fab8D3^FcRn–^ accumulated at high concentrations around brain deposits of Aβ already hours after administration.^14^ The combination of high brain uptake and fast blood clearance observed for Bapi-Fab8D3^FcRn–^ makes it a potential candidate for same-day immunoPET imaging. Thus, in the current study, the antibodies were radiolabeled with fluorine-18 to first compare their in vivo brain pharmacokinetics with PET and second to assess the ability of Bapi-Fab8D3^FcRn–^ to quantify Aβ pathology in Aβ-expressing mice on the same day as administration. Results from the current study may have important implications for the understanding of bispecific Aβ antibody brain distribution and for the development as companion diagnostics to therapeutic bispecific antibodies, such as trontinemab, that are currently under clinical evaluation.

## Results and Discussion

### Antibody Radiolabeling

The monoclonal, humanized Aβ antibody Bapi^FcRn–^ and its bispecific, brain-penetrating variant Bapi-Fab8D3^FcRn–^, both with reduced binding to FcRn (Figure 1A), were produced as previously described.^14^ For fluorine-18 labeling, the antibodies were first functionalized with a trans-cyclooctene (TCO) moiety to enable a specific tetrazine ligation click reaction with a fluorine-18 labeled tetrazine molecule, [^18^F]­Tz, to attach the radiolabel to the antibody.^13^ [^18^F]­Tz was produced with a molar activity of 82.2 ± 0.3 GBq/μmol. To calculate the average number of TCO molecules per antibody, an excess of [^18^F]­Tz was added to TCO-Bapi-Fab8D3^FcRn–^. After gel electrophoresis, quantification of total [^18^F]­Tz as well as [^18^F]­Tz in the high-molecular-weight fraction were used to calculate the TCO/antibody ratio, which was estimated to be 3.5 (Figures 1B and S1). The tetrazine ligation used to radiolabel the antibodies was well tolerated, resulting in pure antibody preparations with minimal aggregation, as assessed with size exclusion chromatography (SEC) (Figure S2). ELISA analysis showed retained binding of both antibodies to Aβ after TCO-modification and after reacting with the fluorine-18 labeled tetrazine (Figure 1C,D). Bapi-Fab8D3^FcRn–^ modified with TCO and labeled with fluorine-18 also showed retained binding to TfR (Figure 1E). This two-step radiolabeling method has the advantage that the antibody can be functionalized in large quantities and quality tested prior to radiolabeling, which allows a standardized labeling procedure. In addition, the fast kinetics of the TCO-tetrazine ligation requires only a short incubation and purification to obtain the final radiolabeled antibody.

**Figure 1:**
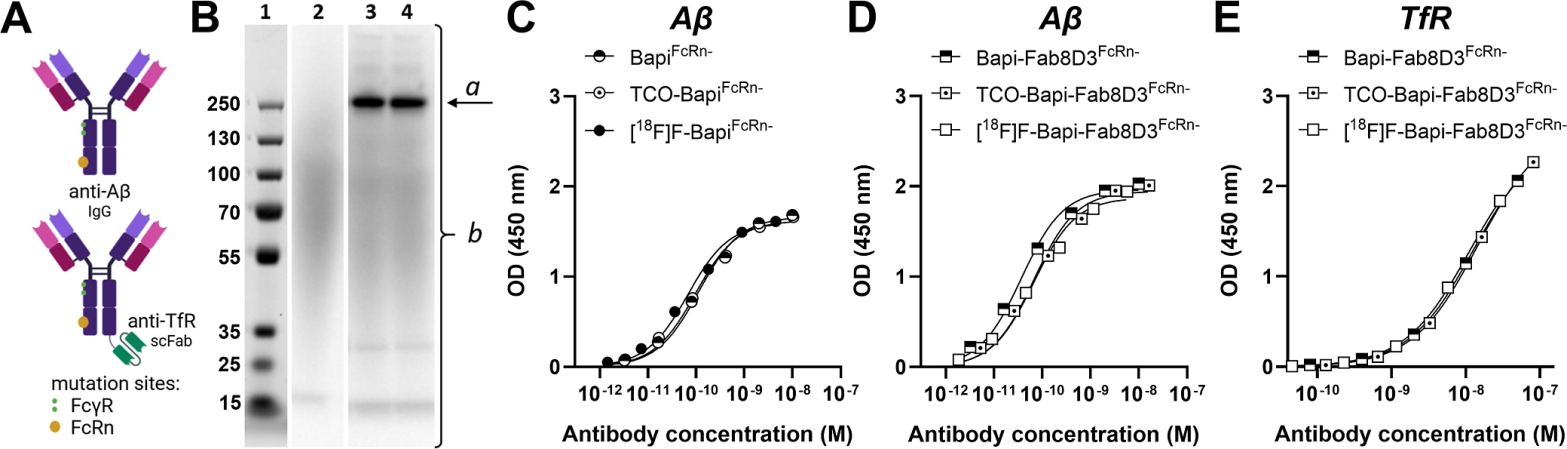
Antibody modification, radiolabeling, and quality control. (A) Antibodies used in the present study, Bapi^FcRn–^ (upper) and Bapi-Fab8D3^FcRn–^ (lower), both carrying mutations to prevent interactions with Fc gamma receptors (FcγR) and the neonatal Fc receptor (FcRn) (created with Biorender). (B) Number of TCOs per antibody molecule was quantified as the fraction of activity associated with the antibody (a/b) divided by the antibody/[^18^F]­Tz concentration ratio. Lane 1—MW standard; lane 2—[^18^F]­Tz; lane 3—[^18^F]­Tz + TCO-Bapi-Fab8D3^FcRn–^; lane 4—[^18^F]­Tz + TCO-Bapi-Fab8D3^FcRn–^. Aβ binding of Bapi^FcRn–^ (C) and Bapi-Fab8D3^FcRn–^ (D) as well as TfR binding of Bapi-Fab8D3^FcRn–^ (E) was assessed with indirect ELISA, comparing nonmodified with TCO-modified and fluorine-18 labeled antibody.

### PET Imaging of Antibody Pharmacokinetics

Groups of WT mice were injected with either [^18^F]­F-Bapi^FcRn–^ or [^18^F]­F-Bapi-8D3^FcRn–^. Subsequently, the mice underwent alternating PET scanning (Figure 2A and Table S1) to obtain an almost complete visualization of the total brain concentration over 9 h after antibody administration. PET images obtained already at 0–5 min after injection showed a difference in brain concentration between the two antibodies, with higher brain uptake of the bispecific [^18^F]­F-Bapi-8D3^FcRn–^ compared to that of [^18^F]­F-Bapi^FcRn–^. The difference in brain concentration between the two antibodies was also seen in PET images at all later investigated time points throughout the 9 h scan period (Figure 2B).

**Figure 2:**
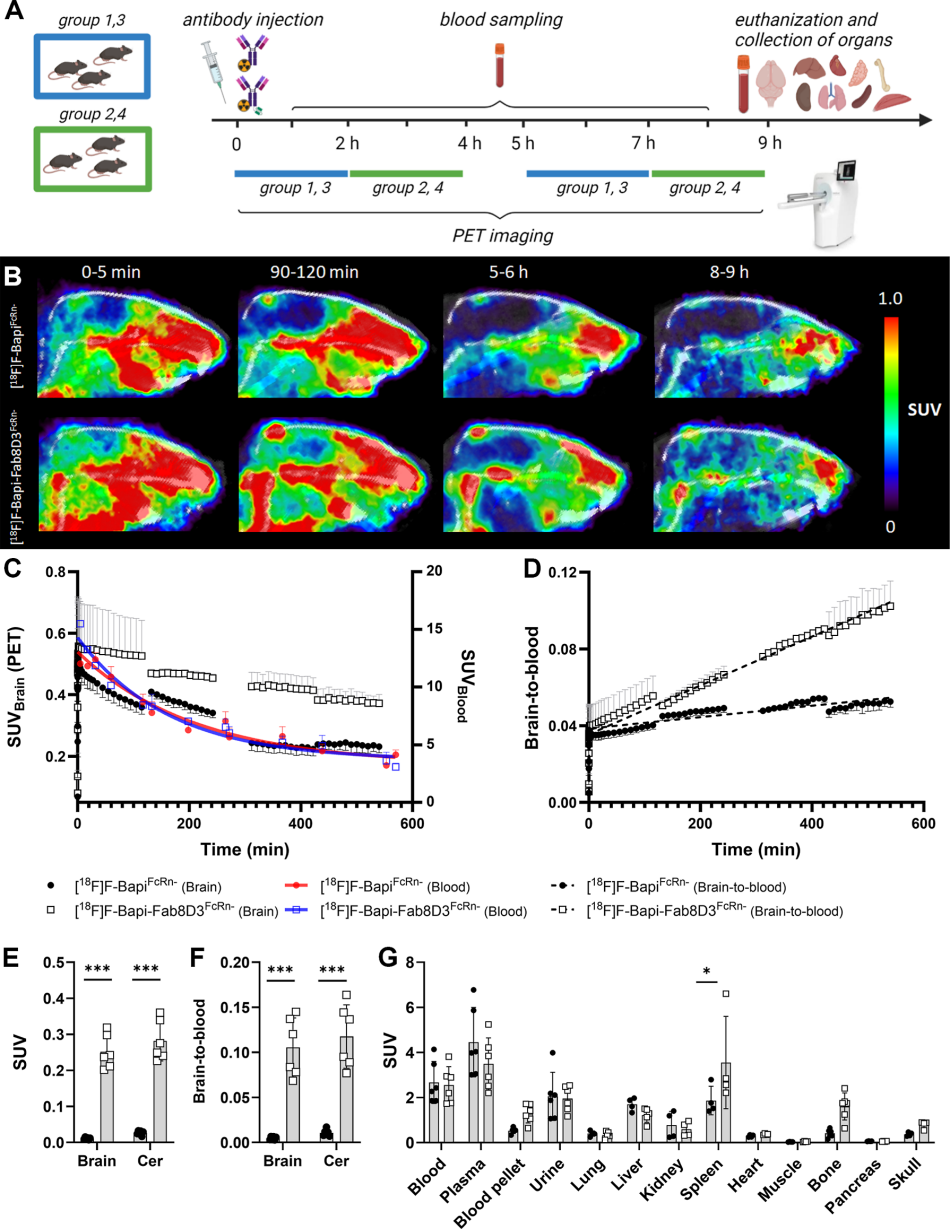
Pharmacokinetics of [^18^F]­F-Bapi^FcRn–^ and [^18^F]­F-Bapi-Fab8D3^FcRn–^ in WT mice. (A) Schematic description of the PET study design (created with Biorender). (B) Representative summed PET images obtained at four different time points (0–5 min, 90–120 min, 4–5 h, and 8–9 h) after administration of [^18^F]­F-Bapi^FcRn–^ and [^18^F]­F-Bapi-Fab8D3^FcRn–^ to WT mice. (C) Time–activity curves of PET scans in (B) and blood concentrations of [^18^F]­F-Bapi^FcRn–^ (red) and [^18^F]­F-Bapi-Fab8D3^FcRn–^ (blue) are displayed in the same graph (D). Brain-to-blood ratio, based on in individual brain concentrations (SUV) (from C) and estimated blood concentrations during the PET scans derived from blood samples taken over the course of the experiment (individual blood curves displayed in Figure S3). Dashed lines represent nonlinear regression model for the brain-to-blood ratio of the respective antibody. (E) *Post mortem* brain concentrations of [^18^F]­F-Bapi^FcRn–^ and [^18^F]­F-Bapi-Fab8D3^FcRn–^ in PET scanned WT mice, expressed as SUV. (F) Brain-to-blood ratio of (E). (G) *Post mortem* biodistribution of [^18^F]­F-Bapi^FcRn–^ and [^18^F]­F-Bapi-Fab8D3^FcRn–^ in peripheral organs of PET scanned WT mice.

Whole brain antibody concentrations were obtained by quantification of the PET images and plotted together with the blood concentration curves for both antibodies (Figure 2C). The brain concentration of [^18^F]­F-Bapi^FcRn–^ declined immediately after injection at a rate similar to the elimination from blood, with a final brain concentration of 0.23 ± 0.03 (SUV) at 9 h postinjection (Figure 2C). In contrast, [^18^F]­F-Bapi-Fab8D3^FcRn–^ displayed a slight increase during the first minutes and then a stable signal over the following 2 h at a substantially higher level than [^18^F]­F-Bapi^FcRn–^. After a significant drop in brain concentration between the first and second scan, the [^18^F]­F-Bapi-Fab8D3^FcRn–^ concentration stayed relatively stable over the remaining scanning time, with a final brain concentration of 0.39 ± 0.01 (SUV) at 9 h post injection (Figure 2C). Normally, a TfR binding bispecific antibody has a much faster elimination from blood, compared with its corresponding regular IgG format, which prevents a fair comparison of the brain pharmacokinetics of these antibody formats.^15,16^ However, here, [^18^F]­F-Bapi^FcRn–^ and [^18^F]­F-Bapi-Fab8D3^FcRn–^ showed almost identical elimination rates from blood due to the mutation in the FcRn-binding domain, which also substantially reduced the half-life of both antibodies (Figure 2C). The almost identical blood concentration curves of [^18^F]­F-Bapi^FcRn–^ and [^18^F]­F-Bapi-Fab8D3^FcRn–^ allow for a direct comparison of antibody brain exposure relative to blood, expressed as a brain-to-blood ratio.

A continuous brain-to-blood ratio was calculated from the PET-derived brain concentrations, divided by blood concentrations of individual mice, estimated from blood samples taken during the experiment. The brain-to-blood ratio of [^18^F]­F-Bapi^FcRn–^ remained almost stable at around 4%, a number generally used as an estimation of the blood volume of the brain, suggesting that the antibody displayed no or very low brain accumulation over 9 h of PET scanning. In contrast, the brain-to-blood ratio of [^18^F]­F-Bapi-Fab8D3^FcRn–^ increased steadily with time, clearly displaying its accumulation in the brain (Figure 2D). Similar to the brain concentration displayed in Figure 2C, a significant drop in the brain-to-blood ratio of [^18^F]­F-Bapi-Fab8D3^FcRn–^ was seen between scan 1 and scan 2. As no significant difference in blood concentration was observed between the animals of the first and second scan or between antibodies 2 h post injection (Figure S4), it can probably be ruled out that this drop was caused by a difference in antibody clearance. Instead, we speculate that the higher brain-to-blood ratio originates solely from the brain, potentially due to anesthesia-induced alterations in TfR trafficking at the BBB. The active BBB transport of [^18^F]­F-Bapi-Fab8D3^FcRn–^ observed in the PET-based quantification of brain concentrations was also reflected in the ex vivo measured brain concentration of the antibodies 9 h after injection. Thus, in perfused brains devoid of blood, a substantial amount of [^18^F]­F-Bapi-Fab8D3^FcRn–^ remained in the brain, with only very low levels of [^18^F]­F-Bapi^FcRn–^, resulting in about a 10-fold difference between the antibodies both in absolute brain concentration (Figure 2E) and brain-to-blood ratio (Figure 2F). Notably though, the reduced total exposure of bispecific antibodies harboring mutations to reduce FcRn interactions obtained a lower total brain uptake compared to nonmutated variants, as previously reported.^14,17^ Ex vivo biodistribution to peripheral organs was in general similar for the two antibodies, mostly reflecting the antibody concentration in blood. However, the spleen and bone, as well as the blood cell pellet, where TfR-binding antibodies are typically retained due to specific interactions, displayed higher [^18^F]­F-Bapi-Fab8D3^FcRn–^ concentrations than [^18^F]­F-Bapi^FcRn–^ concentrations (Figure 2G).

To study antibody brain pharmacokinetics in vivo is challenging. We have previously demonstrated the active transport of bispecific TfR-binding antibodies in WT mice over a period of several hours with in vivo microdialysis.^18^ However, although microdialysis gives a time-resolved illustration of the actual intrabrain concentrations of an antibody, it is a focal method that describes what happens in a very limited space. Therefore, PET imaging, which shows the global antibody concentration in the whole brain, serves as a useful and complementary method to study the brain pharmacokinetics of antibodies. Indeed, PET and microdialysis provide similar estimates of the dynamics of antibody brain entry, with a peak around 8 h after administration.^18^ These findings have also been corroborated by ex vivo measurements of antibody concentrations in the brain.^19^

### PET Quantification of Aβ Pathology

The primary aim of developing bispecific antibody tracers is to specifically visualize and quantify intrabrain targets, e.g., Aβ, with PET imaging. We have previously reported that PET imaging with a fluorine-18-labeled bispecific IgG antibody could not discriminate between AD and WT mice 12 h after antibody administration.^13^ To investigate if the improved pharmacokinetic properties of [^18^F]­F-Bapi-Fab8D3^FcRn–^ would enable a better performance in this respect, WT and *App* ^*NL‑G‑F*^ mice (*n* = 3 per group) were administered [^18^F]­F-Bapi-Fab8D3^FcRn–^ and PET scanned 12 h after injection (Table S1, Figures 3A and S5). Quantification of brain concentrations based on acquired PET images indicated a trend toward higher [^18^F]­F-Bapi-Fab8D3^FcRn–^ retention in *App* ^*NL‑G‑F*^ compared to WT mice in whole brain (*p* = 0.10), cortex (*p* = 0.09), and hippocampus (*p* = 0.07). When expressed as a brain-to-blood ratio, variation between animals decreased, and significant differences were seen in all three brain regions (Figure 3B). Ex vivo analysis of brains from PET-scanned mice revealed a significant difference in brain concentrations between *App* ^*NL‑G‑F*^ compared to WT mice (Figure 3C), that was highly correlated with PET quantification of the same brains (Figure 3D). Ex vivo biodistribution to peripheral organs showed a relatively high uptake in spleen and bone due to interaction with TfR in these tissues. The low uptake in skull compared to that in bone suggests this is primarily binding to TfR in bone marrow, which is further supported by small hot spots in the marrow containing upper part of the skull, as seen in the PET images. There were no differences in biodistribution between the *App* ^*NL‑G‑F*^ and WT mice.

**Figure 3:**
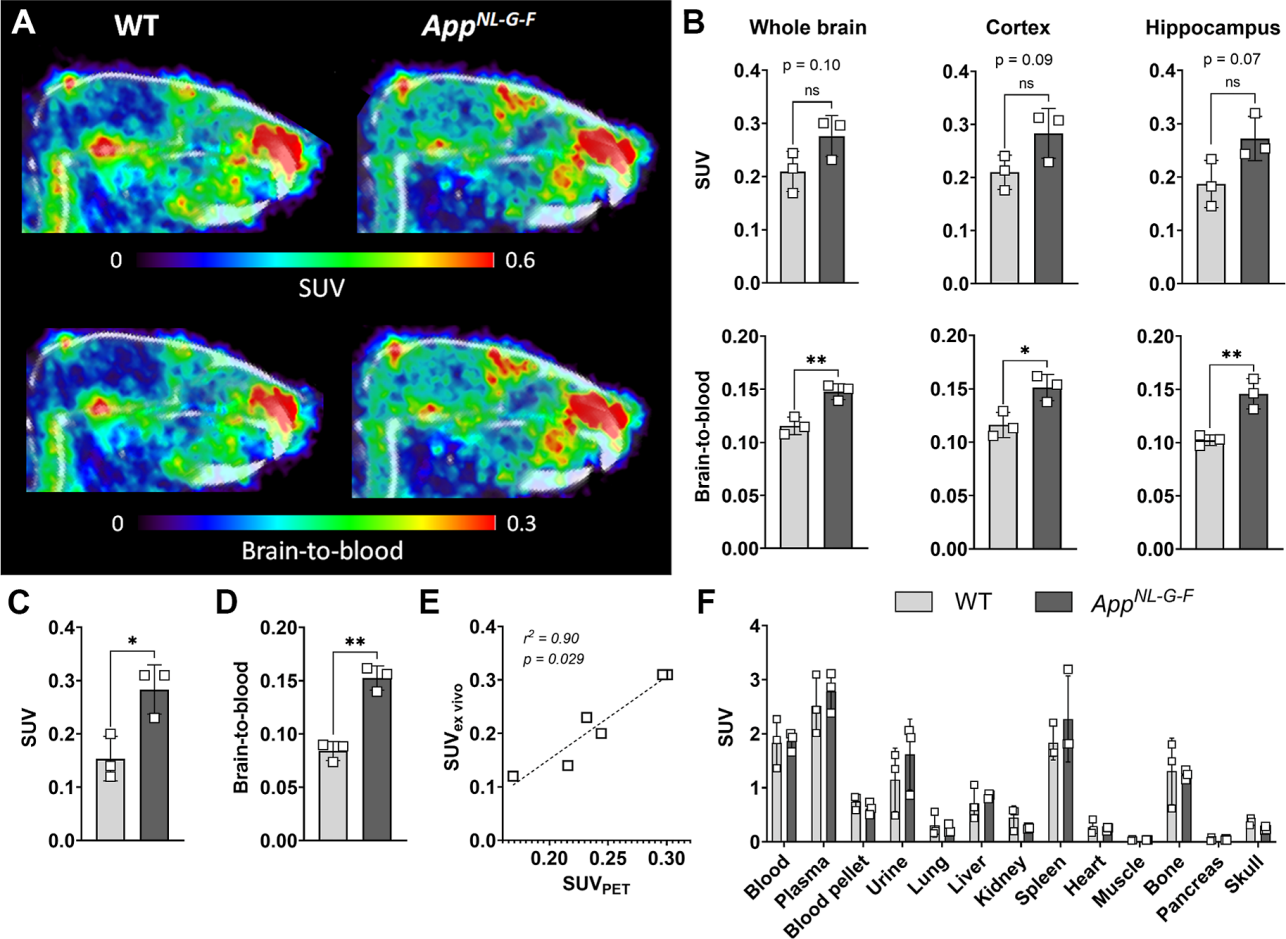
[^18^F]­F-Bapi-Fab8D3^FcRn–^ PET imaging in WT and *App* ^*NL‑G‑F*^ mice. (A) Representative sagittal PET images obtained during a 60 min scan 12 h after the injection of [^18^F]­F-Bapi-Fab8D3^FcRn–^ in WT (*n* = 3) and App^NL‑G‑F^ (*n* = 3) mice. Images are scaled to SUV (upper panel) or brain-to-blood ratio (lower panel). B. Regional quantification of PET images from (A) showing [^18^F]­F-Bapi-Fab8D3^FcRn–^ retention in whole brain (without cerebellum), cortex, and hippocampus, expressed as SUV (upper panel) or brain-to-blood ratio (lower panel). (C) Ex vivo concentrations of [^18^F]­F-Bapi-Fab8D3^FcRn–^ in *post mortem* whole brain (without cerebellum) from PET scanned mice, expressed as SUV. D. Brain-to-blood ratio of [^18^F]­F-Bapi-Fab8D3^FcRn–^ in *post mortem* whole brain of PET scanned mice. (E) Correlation analysis of PET and ex vivo quantification of [^18^F]­F-Bapi-Fab8D3^FcRn–^ concentration in PET-scanned mice 12 h after injection. (F) Ex vivo biodistribution of [^18^F]­F-Bapi-Fab8D3^FcRn–^ in peripheral organs from PET-scanned mice 12 h after injection.

To visualize antibody distribution in brain tissue, ex vivo autoradiography was performed on brain sections from WT and *App* ^*NL‑G‑F*^ mice 12 h after the injection of Bapi-Fab8D3^FcRn–^ radiolabeled with iodine-125. This radionuclide enabled prolonged exposure of the phosphor imaging plate, which was essential to compensate for the low radioactivity emitted by a thin tissue section. This approach was necessary because, unlike whole-brain PET imaging with a fluorine-18-labeled antibody, the tissue section would have produced significantly weaker signals. Compared with the WT brain, the *App* ^*NL‑G‑F*^ brain displayed a substantially higher signal, distributed predominantly in the cortical and central parts of the brain (Figure 4A). Immunostaining with 3D6, the murine version of Bapi, revealed an Aβ brain distribution pattern similar to that of [^125^I]­I-Bapi-Fab8D3^FcRn–^ suggesting that the radioactivity signal is indeed the result of interaction with parenchymal Aβ deposits. Moreover, 3D6 staining did not colocalize with CD31 staining, suggesting low abundance of Aβ deposits in the vasculature (Figure 4B), a phenomenon described as cerebral amyloid angiopathy (CAA). TfR is mainly expressed on capillaries, while CAA is found in larger vessels, suggesting that TfR transport directs the antibody away from CAA. It is thus likely that the [^125^I]­I-Bapi-Fab8D3^FcRn–^ signal is mainly derived from interactions with parenchymal Aβ. These findings are in agreement with a previous study where we have shown that [^125^I]­I-Bapi-Fab8D3^FcRn–^ accumulates in *App* ^*NL‑G‑F*^ brain over time and interacts with Aβ deposits inside the brain, with some antibody accumulation also in the perivascular space around vessels.^14^ Moreover, quantification of Aβ in the brains of PET scanned *App* ^*NL‑G‑F*^ mice showed high levels of insoluble Aβ1–42, accompanied by low levels of insoluble Aβ1–40 and soluble Aβ aggregates (Figure 4C). Analysis of individual mice revealed that Aβ1–42, the most abundant Aβ species, aligned well with whole brain PET quantification of [^18^F]­F-Bapi-Fab8D3^FcRn–^ PET scanned mice (Figure 4D). Taken together, there is strong evidence suggesting that the higher PET signal of *App* ^*NL‑G‑F*^ over WT mice is indeed derived from [^18^F]­F-Bapi-Fab8D3^FcRn–^ binding to intraparenchymal Aβ deposits.

**Figure 4:**
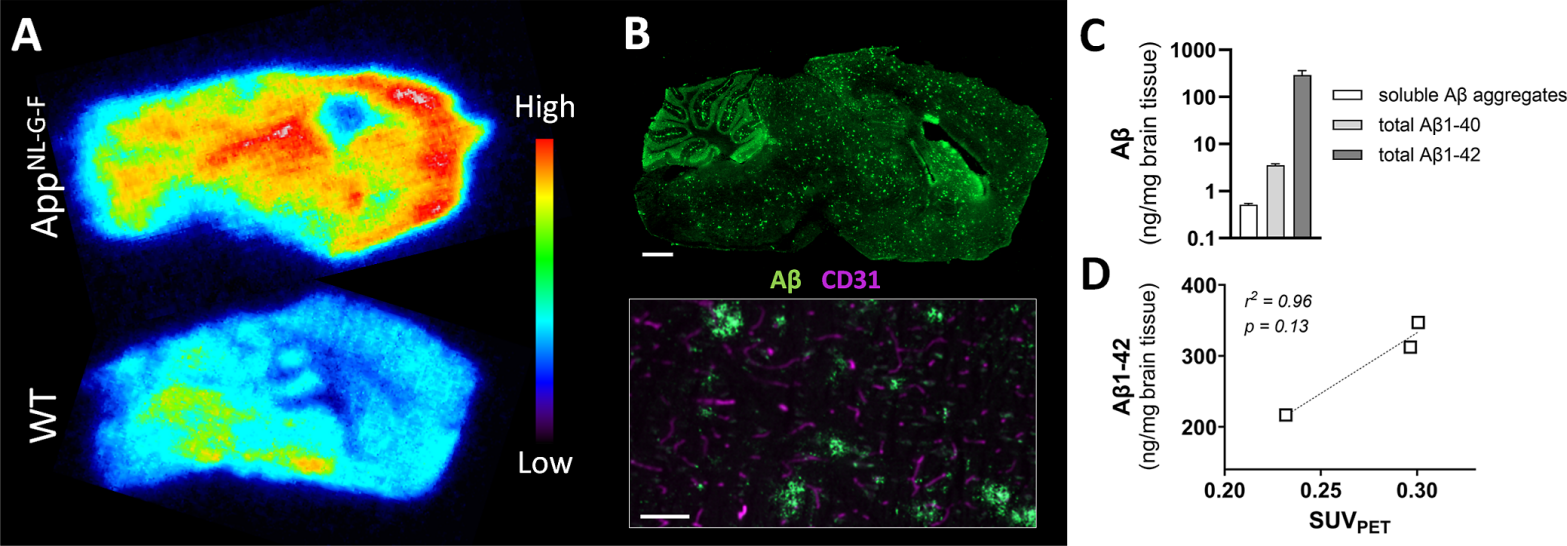
(A) Ex vivo autoradiography of App^NL‑G‑F^ and WT mouse brain, 12 h after injection of [^125^I]­I-Bapi-Fab8D3^FcRn–^. (B) Immunostaining of App^NL–G‑F^ mouse brain with the Aβ antibody 3D6 (murine version of Bapi) alone (upper) and in combination with the vascular marker CD31 (lower). Aβ was found in parenchymal plaques, with very low vascular colocalization. (C) ELISA quantification of TBS soluble Aβ aggregates and of insoluble, total Aβ1–40 and Aβ1–42 in brain extracts from App^NL‑G‑F^ mice that underwent [^18^F]­F-Bapi-Fab8D3^FcRn–^ PET scanning. (D) Correlation of whole brain PET (SUV) and total Aβ1–42 in [^18^F]­F-Bapi-Fab8D3^FcRn–^ PET-scanned App^NL‑G‑F^ mice.

We have previously demonstrated that bispecific antibodies, labeled with iodine-124 for PET imaging at 3–4 days post injection, can be successfully used to quantify Aβ in the brain of AD mice, to follow the progression of Aβ pathology,^11,20^ or to measure the effects of Aβ-lowering treatment.^11,21^ We and others have also demonstrated the successful use of bispecific Aβ antibodies labeled with zirconium-89 for the detection of Aβ pathology in Aβ-expressing mouse models.^17,22–24^ However, the long half-lives of iodine-124 and zirconium-89 increase the radioactive dose administered to the patient. In addition, iodine-124 has limited accessibility and a low proportion of high energy positron decay, which provide suboptimal imaging quality in relation to the administered activity dose. While zirconium-89 is a more accessible and clinically compatible radionuclide, bispecific antibody tracers using TfR-mediated transcytosis for brain uptake tend to produce high background signals when labeled with residualizing radiometals, such as zirconium-89. This phenomenon is caused by TfR-mediated cellular uptake and degradation of the tracer, leaving the radionuclide trapped inside brain cells, where it contributes to a nontarget specific background signal.^17,22–26^ This phenomenon is also observed for antibodies labeled with the radiometal copper-64, which has an intermediate decay half-life of 12.7 h.^22,27^ The use of fluorine-18, with its short half-life and high proportion of low energy positron decay, is thus an attractive alternative to iodine-124 and zirconium-89 as it will reduce the radioactive dose and provide superior image quality. However, the decay half-life of fluorine-18 (∼110 min) sets a tight time limit between injection and imaging. At 12 h after injection, only about 1% of the initial activity remained for detection by the PET scanner. To achieve a high specific signal, it is thus important to rapidly achieve a high brain uptake and fast elimination of unbound tracer from the brain combined with a low signal derived from blood. We have previously shown that fluorine-18-labeled antibodies can be used for PET imaging of Aβ in mice, albeit with a suboptimal contrast, which limits the ability to discriminate between AD and WT mice. In contrast to these previous reports, the current study demonstrates for the first time a full-sized fluorine-18-labeled antibody with pharmacokinetic properties that allow for PET imaging within a time frame that is compatible with fluorine-18. This enabled visualization and quantification of the rapid brain uptake of the TfR-targeted bispecific antibody, in comparison to its regular IgG counterpart. Additionally, despite a lower overall antibody brain uptake of Bapi-Fab8D3^FcRn–^, compared to its nonmutated variant,^14^ the rapid clearance from blood enabled the detection of significant differences in intrabrain Aβ concentrations between WT and *App* ^*NL‑G‑F*^ mice in multiple brain regions already 12 h after antibody injection. Although the number of studied subjects was limited, the significant differences observed between groups along with the strong correlation between PET and ex vivo quantification support the robustness of the results. The low radioactivity remaining at the time of scanning is a limitation, and it remains an open question whether same-day brain PET imaging with antibody tracers will be feasible. Still, this study represents a step toward a clinically compatible immunoPET tracer, whether labeled with fluorine-18, zirconium-89, or copper-64 or using a pretargeted approach,^28^ and we believe that the full IgG bispecific format offers several advantages. First, the possibility to use this antibody format as a companion diagnostic to emerging therapeutic antibodies is attractive; only the FcRn mutation, i.e., a few amino acids of the entire sequence, would have to be introduced. Second, the production and handling of this antibody format are straightforward, with a product that is less sensitive to modifications and radiolabeling, compared to smaller antibody-based constructs and fragments.

## Methods

### Antibody Design and Expression

The antibodies used in this study, Bapi^FcRn–^ and Bapi-Fab8D3^FcRn–^, were designed and expressed as previously reported.^14^ Briefly, they are based on the humanized monoclonal antibody Bapineuzumab (Bapi) that binds specifically to the N-terminus of Aβ (amino acid 1–5).^29^ To generate a brain-penetrating bispecific variant of Bapi, with monovalent binding to the mouse TfR (mTfR), a construct was designed based on a previously published knob-into-hole bispecific format,^30^ where a single Fab fragment of the TfR-binding antibody 8D3 was attached via a peptide linker to the C-terminus of the antibody’s heavy chain.^31^ Both antibodies carry mutations (L234A, L235A, P329G) at the Fc-domain to reduce effector functions.^32^ In addition, Fc mutations (H310A, H435Q) were introduced to reduce binding to the neonatal Fc receptor (FcRn-), which governs the antibodies’ residence time in blood.^33^

The antibodies were synthesized with GeneArt (Thermo Fisher Scientific, Regensburg, Germany), subcloned into the pcDNA3.4 vector backbone, and produced recombinantly in ExpiCHO cells (Gibco, Cat. A29127) using the ExpiCHO Expression System Kit (Thermo Fisher, Cat. A29133). The bispecific antibody was purified with protein A affinity chromatography followed by size exclusion chromatography to exclude aggregated bispecific antibodies using an ÄKTA chromatography system (Cytiva, Uppsala, Sweden).

### Antibody Functionalization

To enable radiolabeling with fluorine-18 (^18^F), the antibodies were first functionalized with a *trans*-cyclooctene (TCO) moiety, which was then reacted with an ^18^F-labeled tetrazine, [^18^F]­Tz, through the inverse electron-demand Diels–Alder reaction. Bapi^FcRn–^ and Bapi-Fab8D3^FcRn–^, at a concentration of 10 μM in PBS, were supplemented with 30 mM sodium carbonate buffer (1:1 mix of 1 M Na_2_CO_3_ and 1 M NaHCO_3_, pH 8.0) and then reacted with axial TCO-NHS (CP-6016, Conju-Probe, San Diego, USA)^34^ dissolved in dimethyl sulfoxide (DMSO) to a 10 mM concentration at a 15-fold TCO–antibody molar ratio, as previously described.^35^ The solution was incubated for 3 h in the dark while being shaken at 600 rpm and subsequently purified from remaining unreacted TCO with Zeba spin desalting columns (7K MWCO, 0.5 mL, Thermo Fisher) and eluted in PBS. Antibody concentration after TCO-modification was assessed with a spectrophotometer (DS-11, DeNovix).

### Radiochemistry

The synthesis of radiolabeled tetrazine 6-[^18^F]­fluoro-N-(4-(6-methyl-1,2,4,5-tetrazin-3-yl)-benzyl)­nicotinamide ([^18^F]­Tz) was performed as previously described.^13^ Starting from 8 to 15 GBq of [^18^F]­fluoride, the isolated radiochemical yield of [^18^F]­Tz was 18 ± 4%, with a molar activity of 82.2 ± 0.3 GBq/μmol and a radiochemical purity of 91 ± 1.1%. TCO-functionalized antibodies TCO-Bapi^FcRn–^ and TCO-Bapi-Fab8D3^FcRn–^ were then coupled with the labeled tetrazine for 30 min at room temperature in PBS. The resulting labeled antibodies were purified with Zeba spin desalting columns (7K MWCO, 0.5 mL) and were obtained with molar activities of 96 ± 31 MBq/nmol for [^18^F]­F-Bapi^FcRn–^ and 84 ± 26 MBq/nmol for [^18^F]­F-Bapi-Fab8D3^FcRn–^. Radiochemical purity of the final antibody constructs was always above 95%, as assessed with radio-TLC on NP Silica gel 60 F254 (Merck, 1.05554.001) with ethyl acetate:*n*-heptane (4:1) as the mobile phase. The retention factors (Rf) were: [^18^F]­F-Bapi and [^18^F]­F-Bapi-Fab8D3FcRn: Rf = 0, [^18^F]­Tz/Rf = 0.8. The integrity of the antibodies after the tetrazine ligation click reaction was determined by SEC using an ÄKTA Go System (Cytiva, Uppsala, Sweden) equipped with a Superdex 200 Increase 10/300 GL (30 cm × 10 mm, 8.6 μm) SEC column (GE Healthcare Life Sciences). Samples were eluted using 1× phosphate buffer (pH = 7.4) as the mobile phase with a run time of 75 min at 0.5 mL/min. Peak intensity was assessed by using the areas under the curve on the UV channel at 280 nm.

### Quantification of TCO-Loading on the Antibodies

Titration experiments were conducted to quantify the amount of reactive TCOs per TCO-mAb according to a previously published procedure.^36^ TCO-mAb in PBS (10 μL of 0.17 μM solutions) was mixed with [^18^F]­Tz stock (10 μL) containing 2 equiv of Tz per expected amount of TCOs on the TCO-mAbs. The mixed samples were incubated at 600 rpm for 1 h at 37 °C. NuPAGE LDS Sample Buffer (6 μL, NP0007, Invitrogen) was added to each sample, and half of each sample was heated to 70 °C and shaken vigorously for 10 min. Subsequently, samples were applied onto SDS-PAGE gels (Bolt 4 to 12%, Bis-Tris, 1.0 mm, 12-well, NW04120BOX, Invitrogen). SDS-PAGE was run in MOPS-SDS buffer at 200 V for 30 min. Separation was monitored by running a PageRuler Plus Pre-Stained Protein Ladder along with [^18^F]­Tz samples. Gels were exposed to phosphor storage screens and read by a Cyclone Storage Phosphor System (PerkinElmer Inc.). Quantification of plate readings was done with Optiquant software (version 3.00, Packard Instruments Co). Radioactivity found in the section of the gel corresponding to molecular weights of approximately 200 kDa (using PageRuler standards as reference) was considered bound to the antibody. The percentage of the total antibody–bound activity per lane of the SDS-PAGE gel was used to calculate the number of reactive TCOs per mAb, accounting for the radiochemical purity of the [^18^F]­Tz. To visualize the proteins, the gel was stained with InstantBlue Coomassie Protein Stain (ab119211, Abcam) and imaged with an iBright FL1500 Imaging System.

## ELISA

To assess if TCO-modification and fluorine-18-labeling of the antibodies affected their binding to Aβ and TfR, ELISA was performed. Anti-IgG ELISA was used to measure the concentration of TCO-modified and radiolabeled antibodies, using a calibration curve of nonlabeled antibodies for quantification. In brief, 96-well half area plates (Corning) were coated with Aβ (25 nM; Innovagen, Lund, Sweden), TfR (in-house production), and anti-human IgG (1 μg/mL, Mabtech, Nacka Strand, Sweden), then blocked with BSA (1% in PBS), followed by addition of serial dilution of unmodified antibody, TCO-modified antibody, and fluorine-18-labeled antibody. For detection, HRP-conjugated antihuman-IgG F­(ab′)_2_ (Jackson ImmunoResearch Laboratories, West Grove, PA, USA) was used in combination with K-Blue Aqueous TMB substrate (Neogen Corp., Lexington, KY, USA). The signals were read with a spectrophotometer at 450 nm. All dilutions were made in ELISA incubation buffer (PBS, 0.1% BSA, 0.05% Tween-20) and washing was performed with ELISA washing buffer (PBS, 0.1% Tween-20).

## Animals

PET imaging was performed in WT mice (*n* = 15) and in the human APP knock-in model *App* ^*NL‑G‑F*^ (*n* = 3), which express the amyloid precursor protein (APP) with a humanized Aβ domain, harboring the Swedish, Arctic, and Iberian APP mutations.^37^ Both females and males were included at an age of 13 months, when abundant Aβ pathology is present in most areas of the brain. All mice, kept on a C57Bl6 background, were housed with unlimited access to food and water in rooms with a controlled temperature and humidity in an animal facility at Uppsala University. All procedures described were approved by the Uppsala County Animal Ethics Board (5.8.18-20401/2020) and were in accord with the rules and regulations of the Swedish Animal Welfare Agency and complied with the European Communities Council Directive of 22 September 22, 2010 (2010/63/EU).

### PET Imaging and Ex Vivo Analyses

WT mice were anesthetized with 5% sevoflurane and placed in a prone position in a Mediso NanoPET/MR (Muenster, Germany). Anesthesia during PET was maintained at 3–4% sevoflurane in a mix of 50% oxygen and 50% medical air. On average, mice were injected with 0.52 ± 0.17 MBq/g body weight of [^18^F]­F-Bapi or 0.46 ± 0.11 MBq/g body weight of [^18^F]­F-Bapi-Fab8D3^FcRn–^. Three mice were simultaneously scanned. Six animals ([^18^F]­F-Bapi^FcRn–^, *n* = 3; [^18^F]­F-Bapi-Fab8D3^FcRn–^, *n* = 3) were scanned between 0 and 2 h post injection and between 5 and 7 h post injection. Blood samples (8 μL) were collected from the tail vein at 2, 3, 4, and 7 h post injection. Six animals ([^18^F]­F-Bapi^FcRn–^, *n* = 3; [^18^F]­F-Bapi-Fab8D3^FcRn–^, *n* = 3) were scanned between 2 and 4 h post injection and between 7 and 9 h post injection. In a second PET experiment, WT (n = 3) and *App* ^*NL‑G‑F*^ (n = 3) mice were injected with 0.84 ± 0.20 MBq/g body of [^18^F]­F-Bapi-Fab8D3^FcRn–^ and PET scanned between 11.5 and 12.5 h after injection. Blood samples (8 μL) were collected from the tail vein of mice at 5 min, 15 min, 30 min, 1, 2, 4, 6, and 9 h post injection. All PET scans were followed by a CT examination for 5 min. One of the [^18^F]­F-Bapi-8D3^FcRn–^ injected mice had to be excluded for technical reasons.

After the last PET scan, all mice were euthanized through cardiac puncture followed by transcardial perfusion with 50 mL of physiological saline for 3 min. Terminal blood was collected and separated into plasma and blood cell pellet. The brain was isolated and divided into the right and left hemispheres. The left hemisphere was further divided into the cerebrum (referred to as brain) and cerebellum. Heart, liver, lung, kidney, spleen, pancreas, femoral bone, and muscle were also isolated. Radioactivity in blood samples and isolated organs was measured with a well counter (GE Healthcare, Uppsala, Sweden). The organ and blood radioactivity concentrations, expressed as standard uptake value (SUV) were calculated as following.

SUV = measured radioactivity per gram tissue/injected radioactivity per mouse body weight in gram

The PET data was reconstructed on a 160 × 160 × 128 grid with 0.5 × 0.5 × 0.6 mm^3^ voxels using 3-dimensional ordered-subsets expectation maximization (20 iterations) into the following frames: 10 × 2 s, 2 × 5 s, 3 × 10 s, 2 × 30 s, 3 × 1 min, 5 × 5 min, and 3 × 10 min for the first scan and 12 × 10 min for the remaining three scans. The CT raw files were reconstructed using filtered back-projection. Processing of the PET and CT images was performed with AMIDE version 1.0.423.^38^ The CT scans were manually aligned with a T2-weighted, MRI-based mouse brain atlas^39^ containing outlined regions of interest. The PET images were subsequently aligned with the CT image containing the brain atlas. Time-activity curves (TACs), based on measured concentrations expressed as SUV, were extracted for the whole brain. PET data were further combined with individual blood data to obtain a continuous brain-to-blood ratio over the whole PET scan time.

### Autoradiography

Ex vivo autoradiography was performed to visualize the brain distribution of Bapi-Fab8D3^FcRn–^, radiolabeled with iodine-125 as previously described.^14^ Brain cryosections (20 μm) were prepared from 13 month-old WT and *App^NL‑G‑F^* mice, 12 h after injection of [^125^I]­I-Bapi-Fab8D3^FcRn–^ at a dose of 0.05 MBq/g body weight, and placed in a cassette with a phosphor imaging screen (Fujifilm, Tokyo, Japan) for a 1 week exposure. The plate was scanned on an AmershamTM TyphoonTM Biomolecular Imager (GE Healthcare, Illinois, United States) at 600 dots per inch, and the generated digital image was converted with the Royal Lookup Table in ImageJ.

### Aβ and CD31 Immunostaining

Sagittal 20 μm brain cryosections from a 13 month-old *App^NL-G‑F^* mouse were fixed for 10 min in ice-cold methanol, washed with PBS, and blocked with mouse-on-mouse IgG blocking reagent. The sections were incubated 5 min with PBS 0.1% Tween-20, then with primary antibodies 3D6 (in-house produced) and rat-antimouse CD31 (BD, #553370) overnight at 4 °C with slow shaking. The following day, sections were washed with PBS and incubated 1 h with goat-antimouse (Alexa 488) and goat-antimouse (Alexa 647) secondary antibodies, mounted with DAPI and visualized with a Zeiss Observer Z.1 microscope using ZEN software (Carl Zeiss Microimaging GmbH, Jena, Germany).

### Aβ ELISA

For quantification of Aβ in the brains of PET-scanned mice, the left cerebrum was sequentially extracted in TBS and formic acid as previously described.^15^ Soluble Aβ aggregates were quantified by coating a half area 96-well plate with antibody 3D6 (1 μg/mL; produced in-house), followed by blocking with 1% BSA in PBS. TBS brain extracts (diluted 1:50) were incubated overnight at +4 °C and then detected by with 3D6-biotin (0.5 μg/mL: produced in-house) and streptavidin-HRP (1:4000; Mabtech AB, Nacka Strand, Sweden) and developed with TMB substrate as above. A calibration curve of synthetic Aβ protofibrils were used for quantification.

TBS-insoluble, formic acid-extracted Aβ1–40 and Aβ1–42 were quantified by coating a plate with either anti-Aβ40 (1 μg/mL; Agrisera, custom produced) or anti-Aβ42 (1 μg/mL; Invitrogen) overnight at +4 °C, followed by blocking with 1% BSA in PBS. Formic acid brain extracts were neutralized with 2 M Tris and further diluted in ELISA incubation buffer (Aβ1-40-1:500; Aβ1-42-1:20 000) and then incubated overnight at +4 °C, followed by 3D6-biotin and streptavidin-HRP detection as above. Calibration curves of synthetic Aβ1-40 and Aβ1-42 were used for quantification.

### Statistical Analyses

Statistical analyses were performed using the Prism 10 software (GraphPad Software, Inc., La Jolla, CA, USA). Values are reported as mean ± standard deviation or standard error of the mean. Data were analyzed using Student’s t-test or one- or two-way analysis of variance (ANOVA) followed by Bonferroni’s post hoc test. (*p < 0.05, **p < 0.01, ***p < 0.001). Brain and blood concentration curves were obtained by nonlinear regression analysis using a one-phase decay model. For individual curves, Y0 was constrained to SUV 14, based on previous experimental data of weight-corrected blood concentrations in mice.

## Supplementary Material



## Notes

Table of animal data and antibody dosing; full SDS-PAGE gel images; SEC chromatogram of antibodies after TCO-modification and Tz ligation reaction; individual blood elimination curves of PET-scanned mice; and whole field of view PET images of WT and *App* ^*NL‑G‑F*^ mice injected with [^18^F]­F-Bapi-Fab8D3^FcRn–^ (PDF)
