# Supplementary material for: Fluorine-18 ImmunoPET Imaging of Antibody Brain Kinetics and Amyloid-Beta Pathology
Source: ACS Pharmacol Transl Sci. 2025 Jul 11;8(8):2804–13. doi: 10.1021/acsptsci.5c00359 (PMC12340626; doi:10.1021/acsptsci.5c00359)
Supplement: Supplementary file 1 [file pt5c00359_si_001.pdf]

Supporting information

Fluorine-18 immunoPET imaging of antibody brain kinetics and amyloid-beta pathology

Eva Schlein[1], Sara Lopes van den Broek[1], Tiffany Dallas[1], Ken G. Andersson[2], Stina Syvänen[1], Jonas Eriksson[3,4], Dag Sehlin[1*]

[1]*Department of Public Health and Caring Sciences, Uppsala University, 751 85 Uppsala, Sweden*
[2]*BioArctic AB, Stockholm, Sweden*
[3]*Department of Medicinal Chemistry, Uppsala University, 751 23 Uppsala, Sweden*
[4]*PET Centre, Uppsala University Hospital, 751 85 Uppsala, Sweden*

*corresponding author

Dag Sehlin
Rudbeck Laboratory
Dag Hammarskjölds väg 20
SE-75185 Uppsala
Sweden
dag.sehlin@uu.se

Table S1. Animal data and antibody dosing

Experiment	**Antibody**	**Animal n** (WT/App^{NL-G-F})	**Animal age** (months)	**Animal weight** (g)	**Inj. act.** (MBq/g)	**Inj. dose** (nmol/kg)
PK PET	[^{18}F]F-Bapi^{FcRn-}	6/0	13.2±0.6	34±4	0.52±0.17	8.0±1.8
	[^{18}F]F-Bapi-Fab8D3^{FcRn-}	6/0	13.0±0.6	31±4	0.46±0.11	8.6±1.2
Aβ PET	[^{18}F]F-Bapi-Fab8D3^{FcRn-}	3/3	13.3±0.7	27±3	0.84±0.20	14.6±0.50

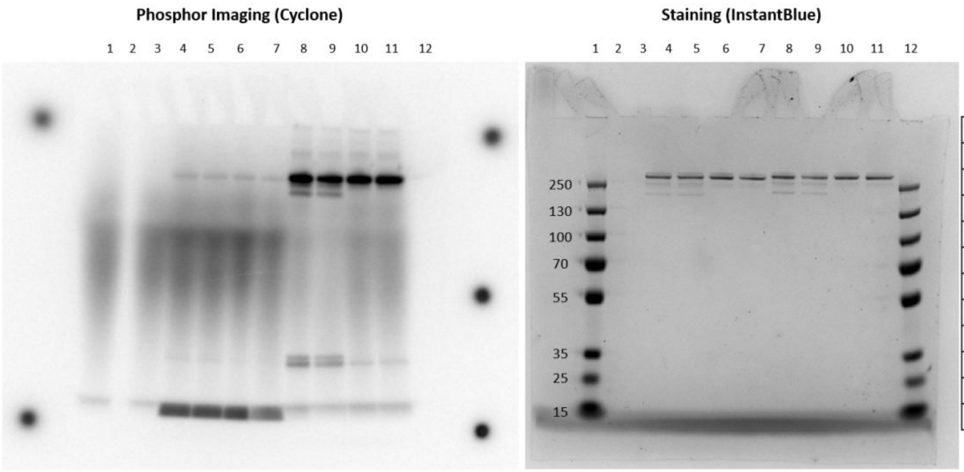

1	$[^{18}F]$Tz
2	MW ladder
3	$[^{18}F]$Tz
4	- (not relevant)
5	- (not relevant)
6	- (not relevant)
7	- (not relevant)
8	- (not relevant)
9	- (not relevant)
10	$[^{18}F]$Tz + TCO-Bapi-Fab8D3^{FcRn-}
11	$[^{18}F]$Tz + TCO-Bapi-Fab8D3^{FcRn-}
12	MW ladder

Figure S1. Full images of the gel shown in Figure 1, analyzed by phosphor imaging (left) and instant blue staining (right). Note that the content of lane 4-9 is not relevant for the present study.

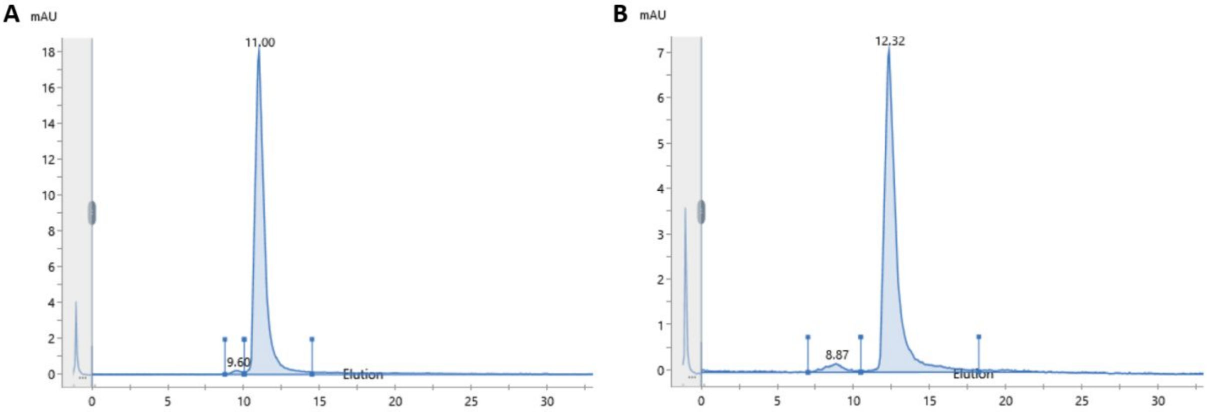

Peak Table

Peak	Retention (ml)	Area (ml*mAU)	Area %
Peak A	9.599	0.1597	1.26
Peak B	10.996	12.52	98.74

Peak Table

Peak	Retention (ml)	Area (ml*mAU)	Area %
Peak A	8.870	0.2771	4.23
Peak B	12.318	6.272	95.77

Figure S2. Size exclusion chromatography (SEC) of **A**. Bapi-Fab8D3^{FcRn-} and **B**. Bapi^{FcRn-} after TCO-modification and tetrazine (Tz) click reaction, demonstrating a monomer content of 99% for Bapi-Fab8D3^{FcRn-} and 96% Bapi^{FcRn-}. The minimal impurity before the main peak may be aggregated antibody.

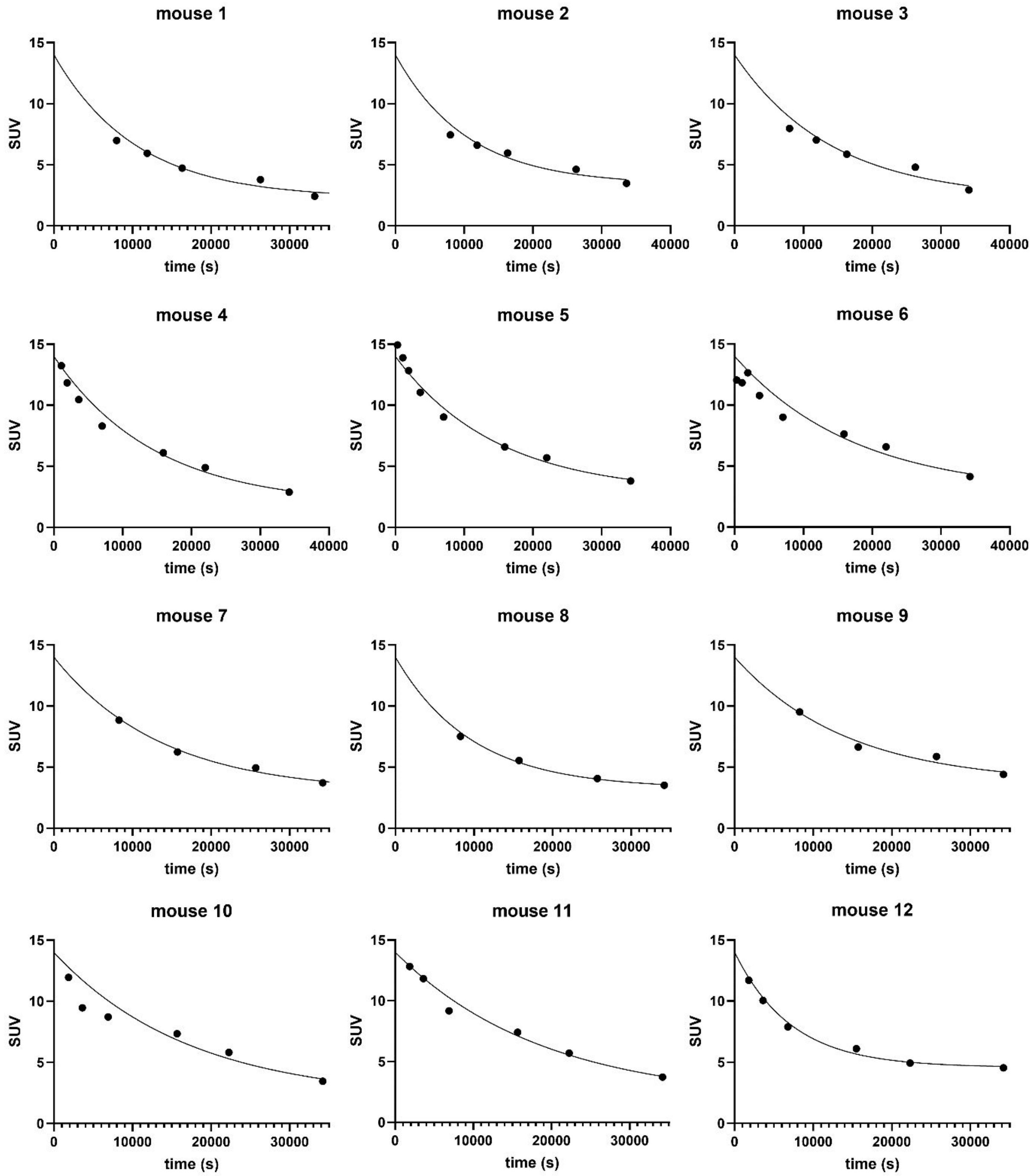

Figure S3. Individual blood elimination curves of 12 PET scanned mice, expressed as SUV. Dots indicate actual blood concentrations measured over the course of the 9 h PET experiment. Elimination curves were calculated with nonlinear regression analysis using a one phase decay model, where Y0 was set to SUV 14, based on previous experimental data of initial weight corrected blood concentrations in mice. Mouse 1-3 were PET scanned as group 1; mouse 4-6 as group 2; mouse 7-9 as group 3 and mouse 10-12 as group 4. Mouse 1, 2, 6, 7, 11 and 12 received [^{18}F]F-Bapi^{FcRn-} while mouse 3, 4, 5, 8, 9 and 10 received [^{18}F]F-Bapi-Fab8D3^{FcRn-}.

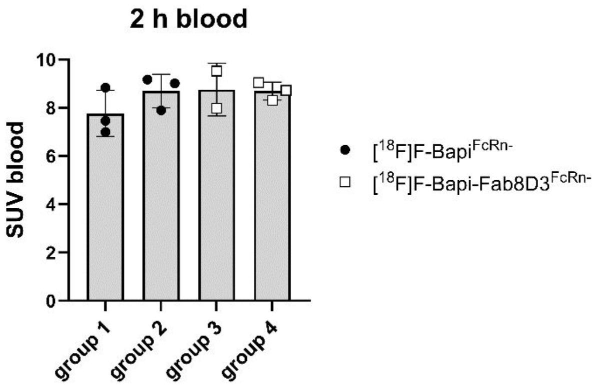

Figure S4. Antibody blood concentration in PET scanned animals, 2 h after injection of [^{18}F]F-Bapi^{FcRn-} or [^{18}F]F-Bapi-Fab8D3^{FcRn-}. Group 1 and 3 were anaesthetized during the first 2 h after injection and then awake, while group 2 and 4 were awake during the first 2 h and then anaesthetized. No difference in blood concentration was seen between mice that were anaesthetized or awake before the 2 h blood sampling.

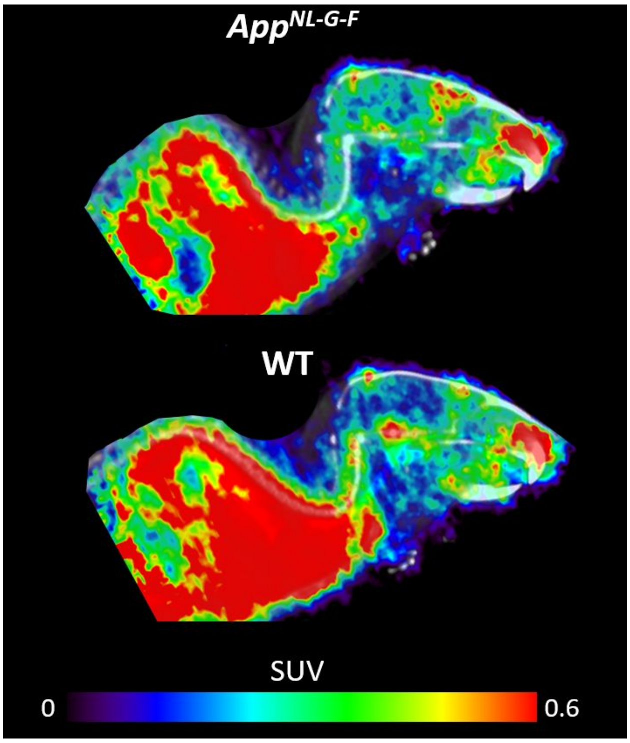

Figure S5. [^{18}F]F-Bapi-Fab8D3^{FcRn-} PET imaging in App^{NL-G-F} and WT mice. Representative sagittal PET images obtained during a 60 min scan 12 h after injection of [^{18}F]F-Bapi-Fab8D3^{FcRn-} in WT (n=3) and App^{NL-G-F} (n=3) mice. Images, scaled to SUV, are the same as shown in Figure 3 and show the entire field of view of the PET scanner.
